# A Novel Smart CFRP Cable Based on Optical Electrical Co-Sensing for Full-Process Prestress Monitoring of Structures

**DOI:** 10.3390/s23115261

**Published:** 2023-06-01

**Authors:** Huanyu Yang, Lian Shao, Jinping Ou, Zhi Zhou

**Affiliations:** 1 School of Civil Engineering, Dalian University of Technology, Dalian 116024, China; yanghy@mail.dlut.edu.cn (H.Y.); shaolian@mail.dlut.edu.cn (L.S.); 2 School of Civil and Environmental Engineering, Harbin Institute of Technology (Shenzhen), Shenzhen 518055, China; oujinping@hit.edu.cn; 3 School of Civil and Architectural Engineering, Hainan University, Haikou 570228, China

**Keywords:** distributed optical fiber sensor (DOFS), coaxial cable fabry–perot interferometer (CCFPI), CFRP cable, anchorage system, prestress monitoring, optical–electrical co-sensing

## Abstract

Carbon-fiber-reinforced polymer (CFRP) is a type of composite material with many superior performances, such as high tensile strength, light weight, corrosion resistance, good fatigue, and creep performance. As a result, CFRP cables have great potential to replace steel cables in prestressed concrete structures. However, the technology to monitor the stress state in real-time throughout the entire life cycle is very important in the application of CFRP cables. Therefore, an optical–electrical co-sensing CFRP cable (OECSCFRP cable) was designed and manufactured in this paper. Firstly, a brief description is outlined for the production technology of the CFRP-DOFS bar, CFRP-CCFPI bar, and CFRP cable anchorage technology. Subsequently, the sensing and mechanical properties of the OECS-CFRP cable were characterized by serious experiments. Finally, the OECS-CFRP cable was used for the prestress monitoring of an unbonded prestressed RC beam to verify the feasibility of the actual structure. The results show that the main static performance indexes of DOFS and CCFPI meet the requirements of civil engineering. In the loading test of the prestressed beam, the OECS-CFRP cable can effectively monitor the cable force and the midspan defection of the beam so as to obtain the stiffness degradation of the prestressed beam under different loads.

## 1. Introduction

Prestressed concrete is widely used in a variety of important infrastructures, including bridges (cable), high-rise buildings, hydraulic buildings, gas storage tanks, etc. Prestressed steel cables and anchorage systems are essential components of prestressed concrete structures, and their performances play a vital role in the safety of the structure. However, the deterioration of the performance of steel cables caused by corrosion seriously affects the safety of these structures [1,2]. Carbon-fiber-reinforced polymer (CFRP) is a kind of composite material with many superior performances, such as high tensile strength, light weight, anti-corrosion, good fatigue, and creep performance [3]. Thus, CFRP cable has great potential to replace steel cable in prestressed concrete structures [4,5,6,7]. However, the CFRP cables are composed of anisotropic tendons. Thus, the tensile strength is high in the fiber direction while radial compressive and shear strength is weak. So, the traditional anchorage systems are not suitable for CFRP cables, which may cause compressive damage to the cable in the anchoring area. Therefore, it is necessary to develop an anchoring system that can give full play to the high tensile strength of CFRP cables.

Over the last thirty years, two main types of CFRP anchoring systems have been developed according to the anchorage principle, i.e., mechanical anchorage and bond-type anchorage [8,9,10,11]. Bond-type anchorage anchors route the CFRP cable through the bond force between the cable and the adhesive. Meier et al. proposed a bond anchorage of CFRP rod-type cable using a conical steel sleeve and a binder made of epoxy resin mixed with Al_2_O_3_ ceramic particles. From the anchoring front to the anchoring end, the Al_2_O_3_ ceramic particle content in the epoxy resin gradually increases, forming a gradient stiffness, reducing the peak stress of CFRP and improving the anchoring efficiency [12]. Fang et al. [13] proposed a multi-rod carbon-fiber-reinforced polymer (CFRP) anchoring system using ultra-high performance reactive powder concrete as a bonding medium and applied it to Aizhai Bridge in China successfully. However, long-term exposure to stress, condensate, temperature difference, acid, alkali, and other complex environments will gradually reduce or even lose the bond between tendons and adhesive [14,15]. The mechanical anchorage relies on the friction between the cable surface and the inner surface of the anchoring member to balance the cable tension. Freyssinet Corp. has developed a wedge fixture anchoring system that uses aluminum sleeves to protect the CFRP bar from being locally crushed by the steel wedge [16]. Zhu et al. [17] proposed a wedge anchoring device with an anchoring efficiency coefficient greater than 90%, which was applied to bridge reinforcement. The mechanical anchorage device is not suitable for multi-bar anchorage, and the sudden change of stiffness between the anchorage device and rods is easy to cause damage to CFRP rods with weak transverse strength. Shao et al. proposed an anchorage system for a single CFRP rod based on cold extrusion [18]. The designed anchorage consists of a steel cylinder and an aluminum sleeve, and an extrusion zone is designed outside the cylinder to produce a suitable contact pressure distribution on the CFRP rod. The aluminum inner sleeve can effectively protect the CFRP rod from being crushed.

The technology to monitor the stress state in real-time during the full life cycle is very important in the application of CFRP cable. Presently, a lot of techniques have been proposed to monitor the stress in the cable, such as vibration methods, impedance-based methods, elasto-magnetic methods, acoustoelastic methods, strain-based methods, and static nondestructive testing (NDT) methods [19,20,21,22,23]. Among them, vibration methods have low sensitivity to prestress force changes, impedance-based methods can only consider prestress force change near the anchorage at present, elasto-magnetic methods are not suitable for in situ monitoring because of low feasibility of instrumentation, acoustoelastic methods also have low sensitivity to prestress force changes and cannot monitor the distribution of the prestressing force along a prestressed concrete structure [24], static NDT methods do not require any direct measure of the tension force in the tendon but require vertical deflections with an accuracy of 0.01 mm, which are not always easy to obtain in situ [25]. In conclusion, strain-based methods show the greatest hope in monitoring prestress, due to the maturity of sensing technology and the good relationship between strain and stress. Due to the relative maturity of strain-based methods in sensors and algorithms, the current research focus is on the universal implementation of strain sensors. Fiber optic sensors have been developed rapidly in recent years and applied to monitor the cable force due to stable sensing characteristics, high accuracy, high-temperature resistance, corrosion resistance, small physical dimensions, low weight, and resistance to electromagnetic interference [26,27]. However, due to the low measurement range and weak impact resistance of optical fiber, the fiber optical sensor cannot monitor the strain entirely until the CFRP cable fails and thereby cannot supervise the state of CFRP cable safety under all loading conditions [28].

This limitation is mainly determined by the material properties of low elongation of optical fiber. Coaxial cables with high elongation (strain up to 15%, more than steel yield deformation) have a similar electromagnetic (EM) theory to optical fiber except for the frequency of the electromagnetic waves that travel within them, which has been greatly developed in recent years. A series of coaxial cable-based sensors have been developed, including coaxial cable Bragg gratings (CCBGs) [29,30,31,32], long-period Bragg gratings (LPBGs) [33], and coaxial cable Fabry–Perot interferometer (CCFPI) [34,35,36]. CCFPI sensor has the advantages of small spatial resolution and adjustable gauge length, which is superior to other coaxial cable sensors. The DOFS (distributed optical fiber sensor) and CCFPI have good complementarity in strain measurement. The DOFS has high accuracy and a small measuring range, whereas the CCFPI has a large measuring range and low precision.

Given the analysis above, an optical–electrical co-sensing CFRP cable (OECS-CFRP cable) was designed and manufactured in this paper. It consists of a new anchorage system and five CFRP bars, including three CFRP-steel wire composite bars, one CFRP-DOFS bar, and one CFRP-CCFPI bar. The new anchorage system is similar to that proposed in Reference [18], except that five CFRP bars can be anchored simultaneously instead of a single rod. The developed OECS-CFRP cable can not only measure the strain of CFRP cable in the normal use stage with high precision but also measure the large deformation of CFRP cable in the load-bearing capacity reduction phase with relatively low precision. Firstly, a brief description is outlined for the production technology of the CFRP-DOFS bar, CFRP-CCFPI bar, and CFRP cable anchorage technology. Subsequently, the sensing and mechanical properties of the OECS-CFRP cable were characterized by serious experiments. Finally, the OECS-CFRP cable was used for the prestress monitoring of an unbonded prestressed RC beam to verify the feasibility of the actual structures.

## 2. Development of OECS-CFRP Cable

### 2.1. Sensing Principle of DOFS and CCFPI

**Figure 1:**
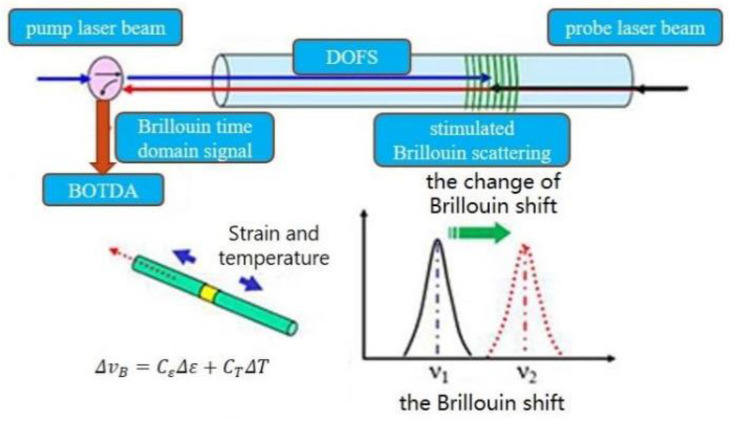
Sensing principle of DOFS.

The DOFS is demodulated by Brillouin optical time domain analysis (BOTDA), which is based on optical time domain reflectometry (OTDR) and stimulated Brillouin scattering (SBS). As shown in Figure 1, the pulsed light and continuous light are injected into the sensing fiber from both ends. The continuous light frequency is tuned, and the Brillouin scattering in the opposite direction will be generated when the frequency difference between the two laser beams is equal to the Brillouin frequency shift. Researchers have shown that the Brillouin shift is linearly dependent on the strain and temperature. The changes in strain and temperature along the optical fiber can be calculated by Equation (1) when the Brillouin frequency shift is obtained using BOTDA [37].

(1) $$\Delta v_{B}=C_{\varepsilon }\Delta \varepsilon +C_{T}\Delta T$$

where Δ*ε* and Δ*T* are the change of strain and temperature, *C_ε_* and *C_T_* are strain and temperature coefficients, and $\Delta v_{B}$ is the change of Brillouin frequency shift.

**Figure 2:**
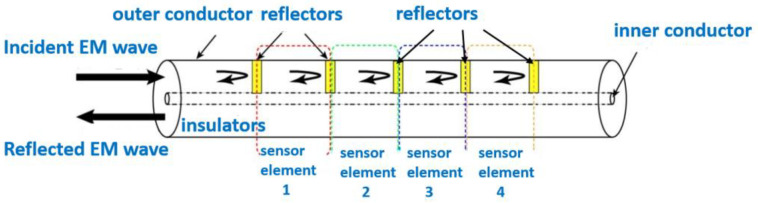
The structure of CCFPI sensor.

**Figure 3:**
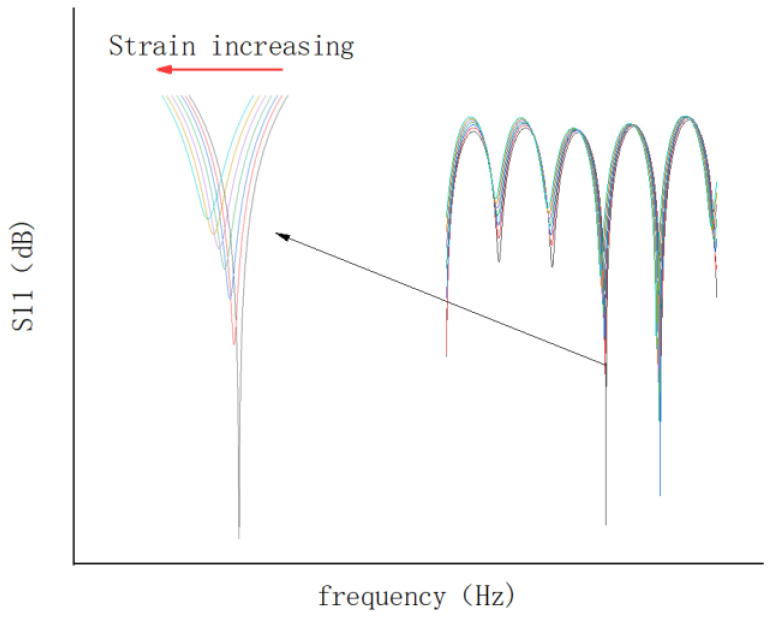
The interferogram of CCFPI sensor.

As shown in Figure 2, the CCFPI sensor is essentially a coaxial cable with *N* (*N* ≥ 2) impedance discontinuities that serve as partial reflectors. The incident electromagnetic wave will be partially reflected at the impedance discontinuities. The two electromagnetic waves reflected at any adjacent reflectors meet each other to generate an interferogram in the spectrum domain. The interferogram has several resonant peaks, as shown in Figure 3. Different color lines represent the interference spectrums caused by different strains. According to Reference [31], the *N*th resonant frequency is shown below:

(2) $$f_{N}=\frac{Nc}{2L\sqrt{\in_{r}}}$$

where *L* is the spacing of reflectors, ∈*_r_* is the relative dielectric constant of the insulating layer, and *c* is the speed of light in a vacuum.

When the cable deforms or the external temperature changes, the physical length or relative permittivity of the cable-insulated layer between the two reflectors will also change accordingly. The change of relative permittivity can be ignored when the measurement accuracy is not strict. The strain value can be obtained directly by measuring the resonant frequency shift as shown in Equation (3):

(3) $$\varepsilon =-\frac{\Delta L}{L}=-\frac{\Delta f_{N}}{f_{N}}$$

According to Equation (3), the relationship between the resonant frequency shift and strain is linear, and the vector network analyzer (VNA) can be used to track the *N*th resonant frequency shift.

### 2.2. Fabrication of CFRP Bars

**Figure 4:**
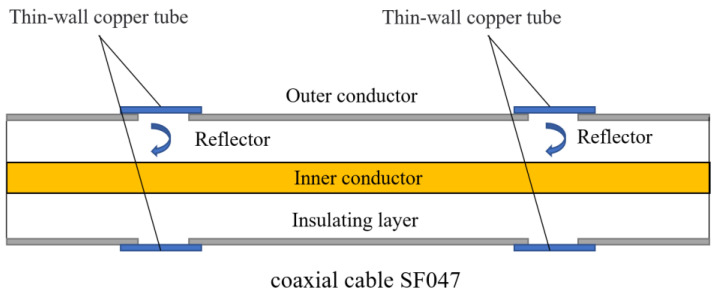
Structural diagram of the CCFPI sensor.

**Figure 5:**
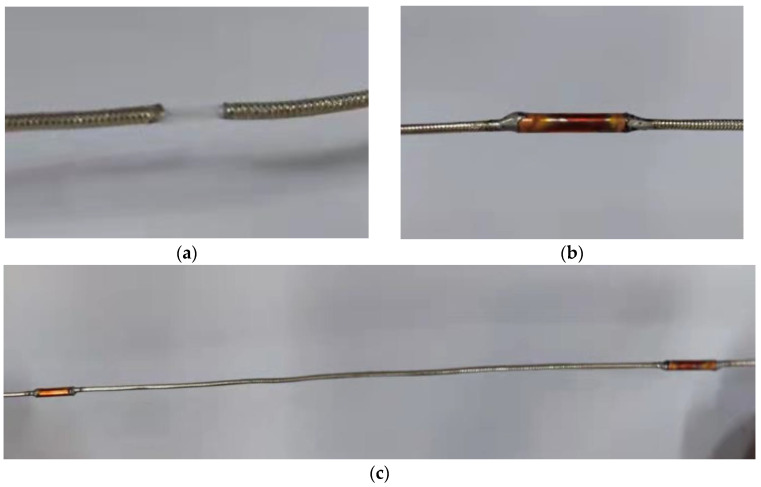
Fabrication process of the CCFPI sensor. (**a**) Peel off the outer conductor; (**b**) sweat the copper tubing; (**c**) the completed CCFPI sensor.

The proposed OECS-CFRP cable consists of three types of CFRP bars with a diameter of 5.2 mm, namely, smart CFRP-DOFS bar, smart CFRP-CCFPI bar, and CFRP-steel wire composite bar. The DOFS was made of common single-mode optical fiber. The high-strength steel wire has a diameter of 0.3 mm, an elasticity modulus of 205 GPa, an ultimate strength of 2300 MPa, and an ultimate elongation of 6%, and accounts for 30.3% of the volume of CFRP-steel wire composite bar. The CCFPI sensor was fabricated using SF047 coaxial cable through the cavity method, as shown in Figure 4 and Figure 5.

The fabrication process of the CCFPI sensor was as follows: (1) A section of SF047 coaxial cable was intercepted, and the positions of two reflectors were marked 20 cm apart; (2) peel off the outer conductor 6 mm at the marked position; (3) the thin-walled copper pipe with an inner diameter of 1.3 mm, a thickness of 0.1 mm and a length of 10 mm is set at the position of the reflector, then the copper pipe and the outer conductor are welded as one with solder paste.

**Figure 6:**
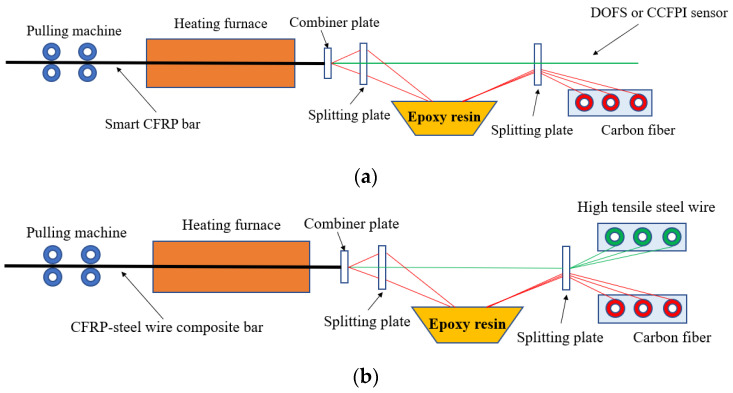
Fabrication process of CFRP bars. (**a**) Smart CFRP bar; (**b**) CFRP-steel wire composite bar.

The manufacturing process of smart CFRP bar and CFRP-steel wire composite bar is pultrusion forming—the same as that of an ordinary CFRP bar—as shown in Figure 6. The pultrusion forming equipment is composed of a splitting plate, combiner plate, resin-wetted tank, heating furnace, and pulling machine. Firstly, the DOFS or CCFPI or high tensile steel wires and carbon fibers are passed through the splitting plate. Then, the DOFS or CCFPI or high tensile steel wires emerged into the carbon fibers through a combiner plate after the carbon fibers passed through the resin-wetted tank. Finally, they were sent together into the heating furnace by the pulling machine, and the CFRP composite bar was solidified at a high temperature of about 200 °C.

### 2.3. Assembly of OECS-CFRP Cable

**Figure 7:**
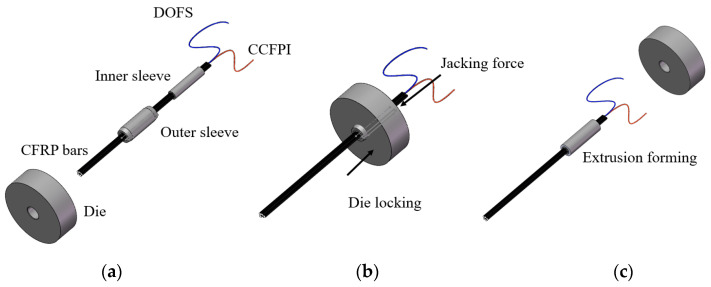

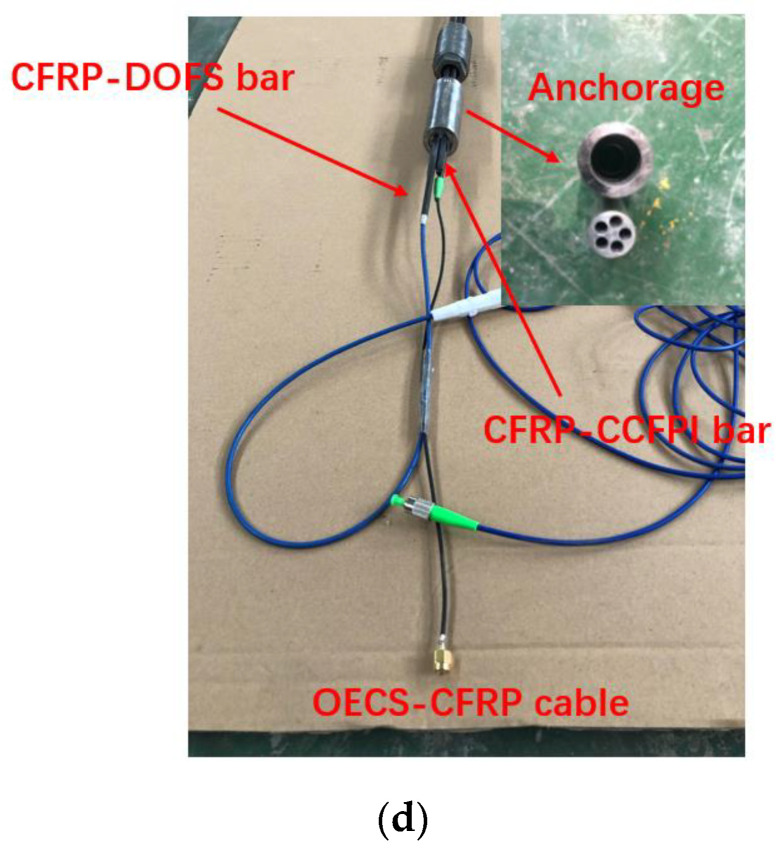
Assembly process of OECS-CFRP cable. (**a**) Anchorage installation; (**b**) extrusion process; (**c**) assembly complete; (**d**) the physical map of OECS-CFRP cable.

The proposed OECS-CFRP cable in this paper is assembled by three CFRP-steel wire composite bars, one CFRP-DOFS bar, and one CFRP-CCFPI bar through the anchoring system, as shown in Figure 7. The anchoring system is developed on the basis of the single CFRP bar anchoring system in Reference [18], which is designed to grip the CFRP bar through radial contact pressure generated by the extrusion process of the metal sleeve. The anchoring system consists of an aluminum inner sleeve and a steel outer sleeve. The inner sleeve has five holes with a diameter of 5.2 mm distributed in the shape of a plum blossom, and the five CFRP bars are passed through the holes, respectively. The CFRP-DOFS bar and the CFRP-CCFPI bar pass through non-adjacent holes. The inner surface of the outer sleeve and the outer surface of the inner sleeve have exactly the same contour so that they can fit closely. As shown in Figure 7a, the CFRP bars, inner sleeve, outer sleeve, and die are put in order before extrusion. During extrusion, the CFRP bars, inner sleeve, and outer sleeve are pushed to go through the die by the extruding machine. In this process, the elastic deformation of CFRP bars and the plastic deformation of metal sleeves occurs. After extrusion, the elastic deformation recovery of CFRP bars is limited by the metal sleeves, which produces radial pressure to grip the CFRP bars tightly. The other end of the CFRP bars is then anchored together in the same way.

## 3. Performance of the OECS-CFRP Cable

### 3.1. Sensing Properties

**Figure 8:**
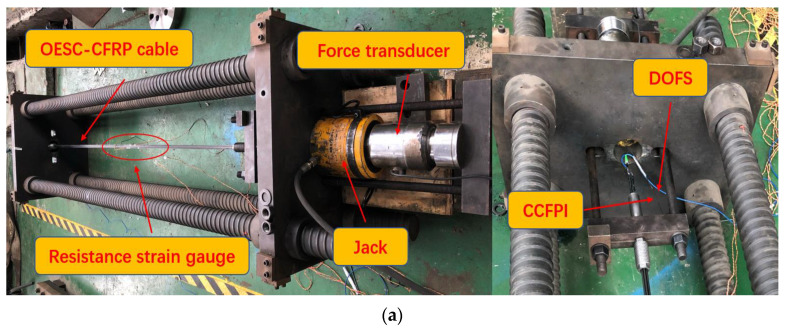

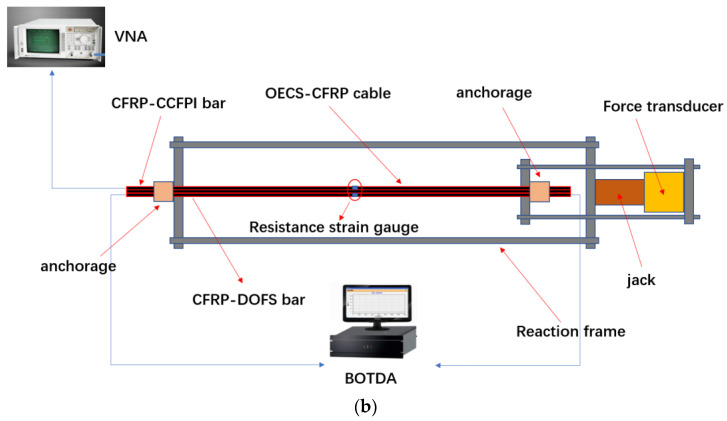
Diagram of sensing performance experiment setup. (**a**) Physical map; (**b**) schematic.

For the sensors, two 2.6 m long OECS-CFRP cables named C1 and C2 with DOFS and CCFPI sensors were prepared, and the experimental setup is shown in Figure 8. The spatial resolution of DOFS and the gauge length of the CCFPI sensor were both 20 cm. An electric resistance strain gage was attached longitudinally to the middle of the CFRP-DOFS bar and the CFRP-CCFPI bar, respectively, whose measurements are used to calibrate the strain sensitivity of the CFRP-DOFS bar and the CFRP-CCFPI bar.

In order to evaluate the force-sensing properties of the OECS-CFRP cable, three groups of cyclic loading and unloading tests were carried out. The tensile force of the OECS-CFRP cable was increased from 0 to 150 kN step by step by 25 kN at a speed of 1 kN/s, then decreased to 0 with the same loading step size and speed. The tension was held for 5 min at each step of loading. During this time, data acquisition was performed.

#### 3.1.1. Strain Sensing Performance of Smart CFRP Bars

**Figure 9:**
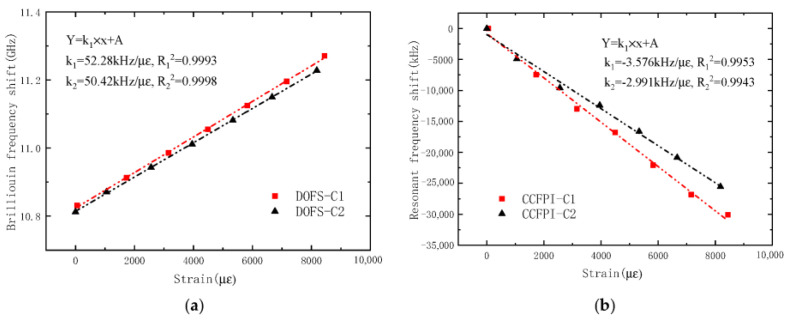
Strain–frequency shift curve of CFRP smart bars. (**a**) DOFS; (**b**) CCFPI.

The strain–frequency shift curve of the smart CFRP bars is shown in Figure 9. It can be seen that the strain of the smart CFRP bars in the two groups of OECS-CFRP cable has a linear relationship with the Brillouin frequency shift or resonant peak frequency shift. The strain sensitivities of CFRP-DOFS bars in the two cables are 52.28 kHz/με and 50.42 kHz/με, respectively, and the square of linear correlation coefficients are above 0.999. The strain sensitivities of CFRP-CCFPI bars in the two cables are −3.576 kHz/με and −2.991 kHz/με, respectively, and the linear correlation coefficient is above 0.994. The test results show that both the CFRP-DOFS bar and CFRP-CCFPI bar have a good self-sensing ability of strain. In comparison, the sensing precision of the CFRP-DOFS bar is higher than that of the CFRP-CCFPI bar.

#### 3.1.2. Tension Sensing Performance of OECS-CFRP Cable

**Figure 10:**
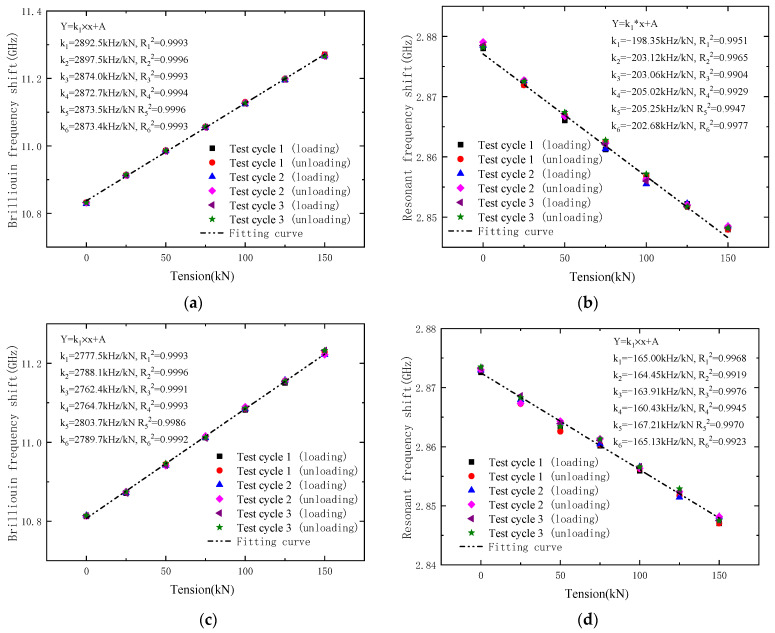
Signal responses of DOFS and CCFPI during three tensile loading cycles. (**a**) DOFS-C1; (**b**) CCFPI-C1; (**c**) DOFS-C2; (**d**) CCFPI-C2.

The tension–frequency shift curves in Figure 10 show the linear relation between the tension and frequency shift of DOFS and CCFPI in the OECS-CFRP cables. As shown in Figure 10a,c, the tension sensitivities of DOFS in specimen C1 vary from 2872.7 to 2897.5 kHz/kN, and the average value is 2880.6 kHz/kN. In specimen C2, they vary from 2762.4 to 2803.7 kHz/kN, and the average value is 2781.0 kHz/kN. It can be seen that the Brillouin frequency shift curves have good consistency, linearity, and no obvious lag.

As shown in Figure 10b,d, the CCFPI resonant frequency shift curves of three groups of cyclic loading and unloading are in good agreement too. The high overlap of data points can be clearly observed in the plots, exhibiting a high linear response and excellent repeatability with no obvious sign of hysteresis at the end of the test. The tension sensitivities of CCFPI in specimen C1 varied from −198.35 to −205.25 kHz/kN; the average value was 202.91 kHz/kN. The average tension sensitivity of CCFPI in specimen C2 is 164.36 kHz/kN. The test results show that the resonant frequency shift curves of CCFPI also have reasonable consistency, linearity, and no obvious lag, but which are all worse than that of DOFS.

According to the Chinese National Standard, “Methods for calculating the main static performance specification of transducers” (GB/T 18459-2001), the main static performance indexes of the OECS-CFRP cable presented in this paper are calculated as shown in Table 1. In Table 1, the maximum linearity of DOFS and CCFPI is 2.64% FS (full-scale) and 7.14% FS, the maximum hysteresis is 1.43% FS and 4.34% FS, the maximum repeatability is 1.25% FS and 4.28% FS, the maximum resolutions are 0.073 kN and 1.217 kN, respectively. It can be seen that the main static performance indexes of DOFS and CCFPI meet the requirements of civil engineering. Compared with CCFPI, DOFS has better linearity, hysteresis and repeatability, and higher resolution.

### 3.2. OECS-CFRP Cable Monitoring from Tensile to Fracture

The tensile damage test of the OECS-CFRP cable was carried out by using the same experimental setup as that of the sensing performance test in Figure 8. A difference from the sensing performance test is that an electric resistance strain gauge was attached longitudinally to the middle of the three CFRP-steel wire composite bars, respectively, instead of the smart CFRP bars. The strain of three CFRP-steel wire composite bars was obtained through electric resistance strain gages; that of the CFRP-DOFS bar and CFRP-CCFPI bar were measured by DOFS and CCFPI sensors, respectively.

**Figure 11:**
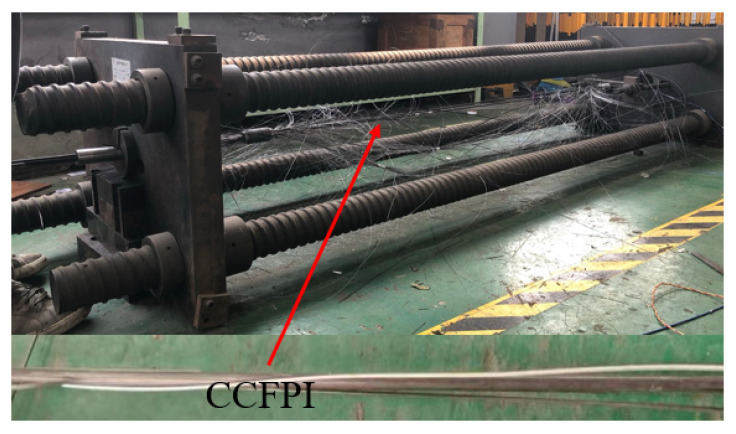
Diagram of OECS-CFRP cable tensile failure.

The load is applied in a step of 25 kN at the beginning of loading and reduced to 5 kN when the tension is greater than 150 kN. The tension of the cable and the strain of the CFRP bars were recorded for each load step or when the cable reached the ultimate tension. The failure of the CFRP cable after reaching the ultimate tension is shown in Figure 11. As can be seen from the figure, the CCFPI sensor is still intact after the CFRP-CCFPI bar splits.

**Figure 12:**
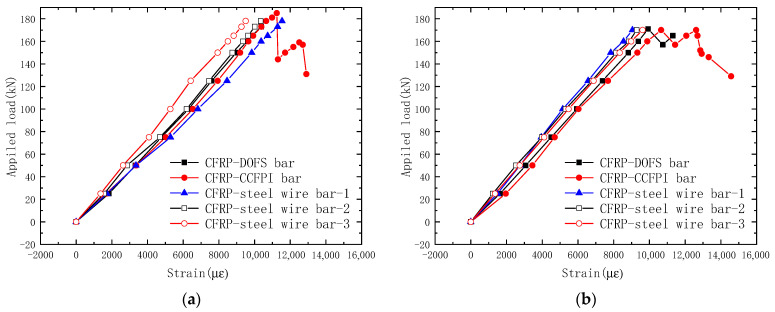
Strain–tension curve of OECS-CFRP cable. (**a**) Specimen C1; (**b**) specimen C2.

Figure 12 shows the relationship between the strain of CFRP bars and the tension of the CFRP cable. It can be seen that the strain of the five CFRP bars increased with the tension increasing. The strains of different CFRP bars in the same CFRP cable are not completely consistent. This is because the tension line does not exactly coincide with the central axis of the CFRP cable, causing the cable to pull eccentrically.

The ultimate tensions of specimens C1 and C2 are 185 kN and 171 kN, respectively. After the CFRP cable reached the ultimate load, the CFRP bars were forced to break one by one, and the tension of the cable gradually decreased. When the tension of the CFRP cable dropped to 75% of the ultimate load, the loading stopped. When the CFRP cable reached the ultimate load-carrying capacity, the carbon fibers burst, and all the strain gauges and DOFS sensor were damaged, and only the CCFPI sensor could still work normally to monitor the deformation of the CFRP cable during the load drop stage, depending on its ability to withstand large deformations and flexibility. As can be seen from the figure, the OECS-CFRP cable does not completely lose bearing capacity after reaching the maximum tensile force and has a certain ductility.

**Figure 13:**
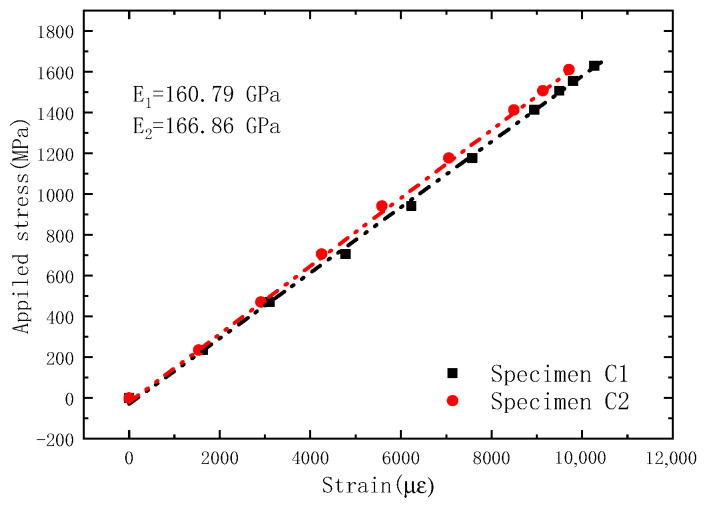
Constitutive relationship of OECS-CFRP cable.

The average strain of five CFRP bars is taken as the strain of CFRP cable, and the ratio of tension of CFRP cable to the total section area of CFRP bars is taken as the stress of the CFRP cable. Figure 13 shows the stress–strain relationship of the OECS-CFRP cable. The elastic modulus of sample C1 and sample C2 are 160.79 GPa and 166.86 GPa, respectively. Taking the average of the measured results of two samples, the elastic modulus of the OECS-CFRP cable is 163.83 GPa and the ultimate strength is 1676 MPa, as shown in Table 2.

### 3.3. Prestressing Monitoring Methods Comparison

We know that the OECS-CFRP cable has the advantages of simple production, easy layout, high sensitivity of cable force perception, and so on from the previous test analysis; in addition, it can monitor the cable failure stage. Table 3 compares the key features of the existing prestress monitoring methods with that of the OECS-CFRP cable proposed in this paper, including spatial distribution (such as point or distributed measurement), accuracy, ambient temperature impact, cost, applicability to CFRP cable, etc.

## 4. Test on Prestress Monitoring of the RC Beam

### 4.1. Design of the Unbonded Prestressed RC Beam

**Figure 14:**
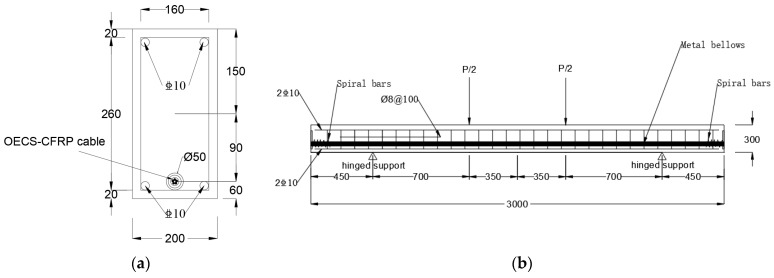
The design of RC beam. (**a**) Cross-section of RC beam; (**b**) elevation view of RC beam.

An unbonded prestressed RC beam of a cross-section of 200 mm × 300 mm and a span of 3000 mm was tested in this research. Figure 14a is the section size and reinforcement diagram of the test beam. Then, four HRB400 steel reinforcements with a diameter of 10 mm were arranged in the compression zone and tension zone of the section. A metallic bellow with a diameter of 50 mm was embedded in RC beams to serve as the prestressed channel. In order to eliminate the stress concentration of the beam, steel plates of 20 mm thickness, 120 mm × 120 mm section and spiral bars of a diameter of 8 mm, spiral inner distance of 110 mm, and five hoops were embedded in the tension and anchorage area of the beam. The mechanical properties of concrete and steel reinforcement are shown in Table 4 and Table 5.

One OECS-CFRP cable proposed in Section 2 was passed through the prestressed channel. The gauge length of the CCFPI sensors was 200 mm. A force transducer was used to measure the tension of the cable.

### 4.2. Tensile Stress Monitoring of the OECS-CFRP Cable

**Figure 15:**
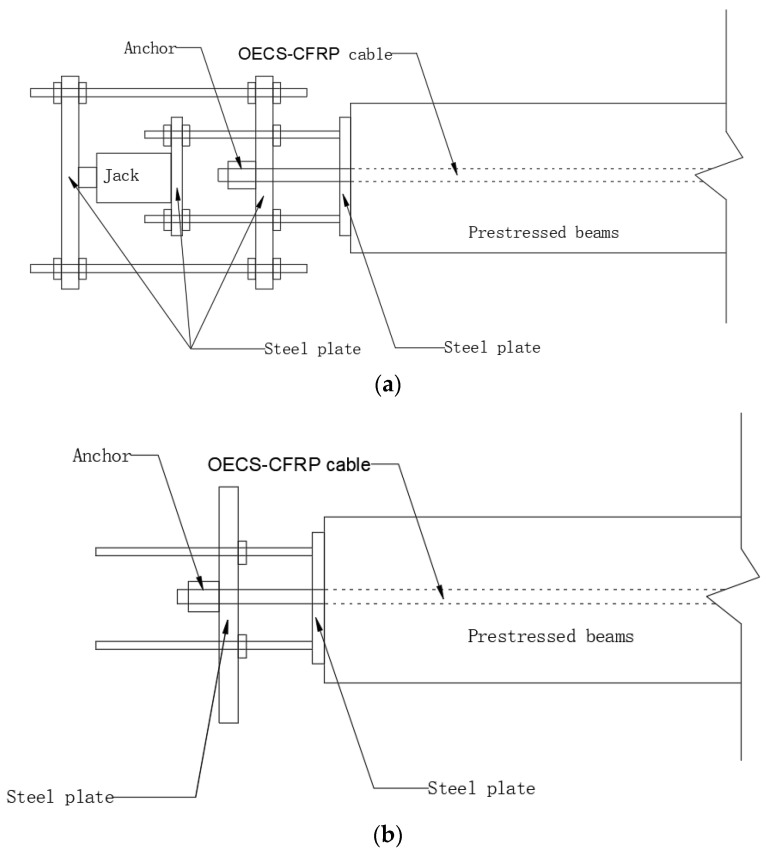
Schematic diagram of prestressed tension of CFRP cable. (**a**) Prestress tension; (**b**) prestress maintenance.

The prestress of the OECS-CFRP cable was applied through a reaction device, as shown in Figure 15. In order to coordinate the strain of CFRP bars in the CFRP cable, pre-tension was required before formal tension, and the pre-tension force was 10 kN. After pre-tension, the strain of each CFRP bar was measured by DOFS, CCFPI, and strain gauges. The limiting bolts on the steel plate were adjusted so that the strain difference between any two CFRP bars was within 10%.

**Figure 16:**
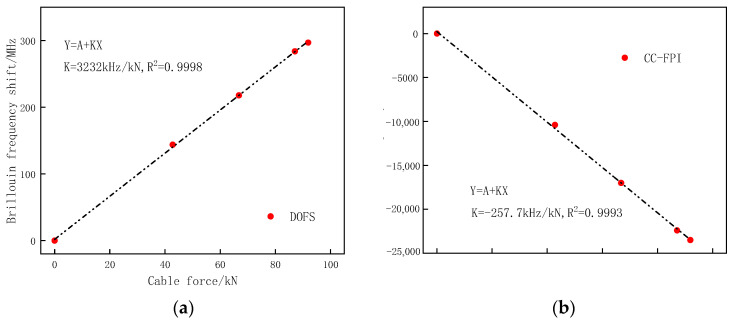
Relationship between frequency shift of DOFS/CCFPI and cable force. (**a**) DOFS; (**b**) CCFPI.

The control tension was set to 91.9 kN; the corresponding tension control stress was 857 MPa. The prestress is loaded in four steps, and the loading grades are 0 kN, 42.8 kN, 66.8 kN, and 91.9 kN, respectively. The tension was held for 10 min at each step. During this time, data acquisition was performed. When the load reaches the control tension, tighten the bolts of the right steel plate to ensure the same degree of tightness so as to prevent the eccentric tension of the CFRP cable. Then unload the jack force to 0; the prestress of the PC beam measured by the force transducer was 87.1 kN. The relationship between the frequency shift of DOFS/CCFPI and cable force is shown in Figure 16.

Figure 16 shows the linear relation between cable force and frequency shift of DOFS and CCFPI in the OECS-CFRP cable. With the tension load increasing gradually, the Brillouin frequency shift of DOFS also increases, and the resonant frequency shift of CCFPI decreases. The force sensitivities of DOFS and CCFPI are 3232 kHz/kN and −257.7 kHz/kN, respectively.

**Figure 17:**
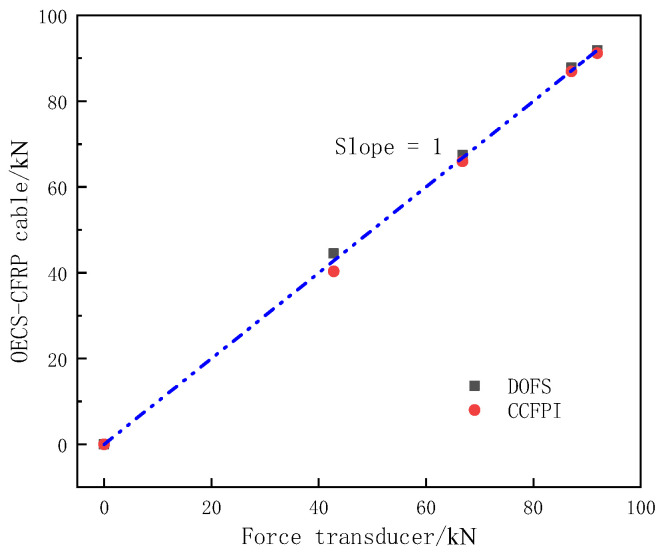
Comparison of the tension measured by the OECS-CFRP cable and force transducer.

A comparison of the tension measured by the OECS-CFRP cable and force transducer is shown in Figure 17. The tension measured by the DOFS and CCFPI is the vertical axis, and the tension measured by the force transducer is the horizontal axis. As shown in Figure 17, the maximum absolute errors between DOFS, CCFPI, and force transducer are 1.70 kN and 2.44 kN, respectively. The maximum relative errors of DOFS and CCFPI are 3.97% and 5.70%. The results show that the OECS-CFRP cable can effectively monitor the tension of the CFRP cable in the process of prestress application.

### 4.3. Loading Test of the Unbonded Prestressed RC Beam

#### 4.3.1. Experiment Design

**Figure 18:**
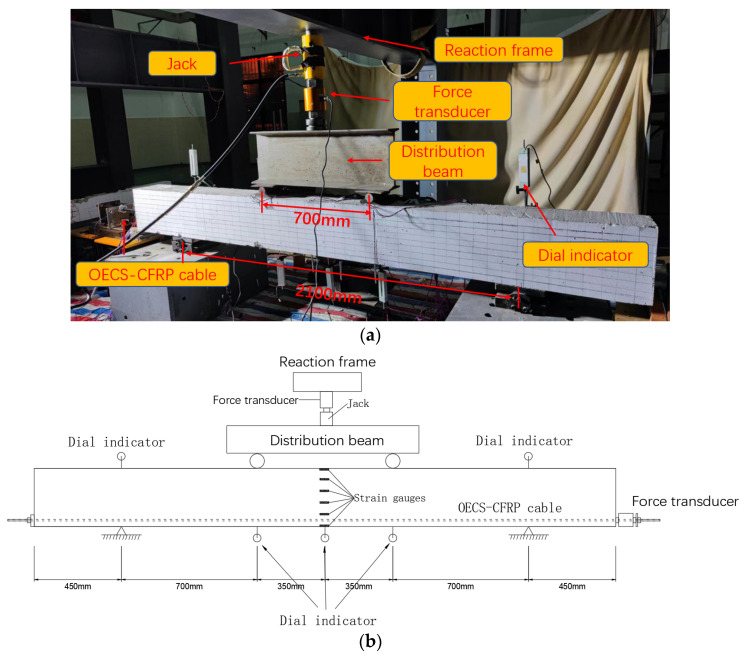
Setup of the loading test. (**a**) Physical map; (**b**) the locations of the measuring points along the specimens.

A four-point bending load test on the unbonded prestressed RC beam was carried out. Figure 18 shows the actual loading setup. The total length of the RC beam is 3000 mm, the distance between the hinge supports on both sides is 2100 mm, and the distance between the two loading points of the distribution beam is 700 mm. One force transducer was installed between the jack and the distribution beam to measure the load in real-time. In addition, another force transducer was installed at the end of the OECS-CFRP cable to monitor the prestress of the PC beam during the four-point bending test. Six strain gauges are uniformly arranged at the midspan of the beam along the beam height direction. A total of five dial indicators are respectively arranged at the hinge supports, the midspan, and two loading points of the beam. The locations of the measuring points along the specimens are shown in Figure 18b. The strain gauges, force transducers, and dial indicators are all demodulated with a sampling frequency of 1 Hz by the static stress–strain test and analysis system DH3818Y. Table 6 lists the performance parameters of the sensors.

At the beginning of loading, increase the load to 40 kN step by step by 20 kN. Then, the load gradually increased step by step by 10 kN until the concrete beam was completely damaged. The load was held for 10 min at each step. During this time, data acquisition was performed.

#### 4.3.2. Finite Element Model

To compare the experimental results, a finite element model (FEM) was established using ABAQUS. To be able to compare with experimental results for validation, the same material properties, dimensions, and prestress value for the PC beam in Figure 18 were applied to the finite element models. The double-broken line model was adopted as the constitutive law of the reinforcements. The plastic damage constitutive model of concrete was derived from the uniaxial tension and compressive stress-strain relations of concrete in Chinese Standard GB50010-2010. The mechanical properties of the OECS-CFRP cable shown in Table 2 were adopted.

**Figure 19:**
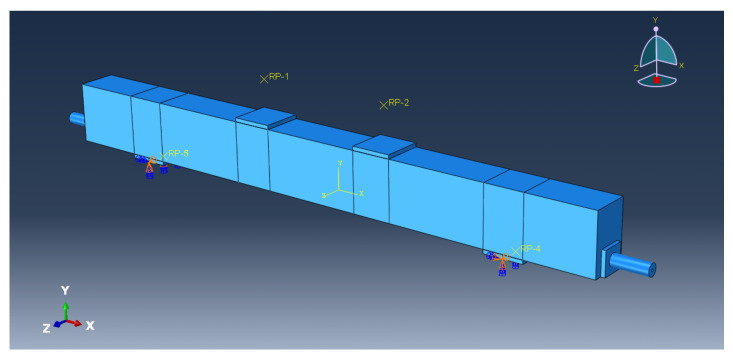
The FEM of the PC beam.

The FEM of the PC beam is shown in Figure 19. The element type of the steel bar is wire, and the rest of the cell type is solid. The boundary condition of the FEM is hinged at both ends. RP-1 and RP-2 are the reference points for coupling constraints with the left and right loading points, respectively. The displacement loading of the PC beam is carried out by applying synchronous vertical displacement to RP-1 and RP-2. The prestress of CFRP cable is simulated by changing its temperature field.

**Figure 20:**
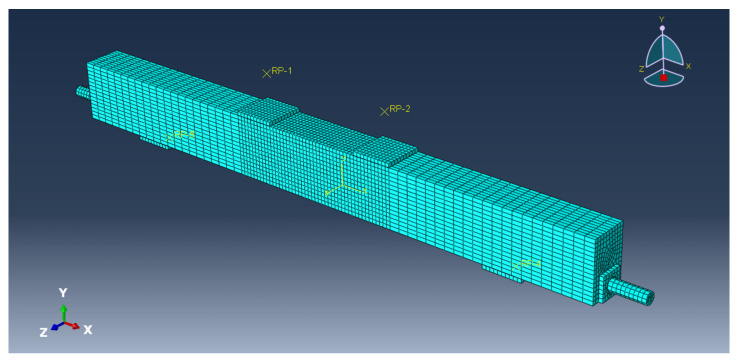
Grid division of the FEM.

The overall mesh size of the FEM is 5 cm, and the mesh size is 1 cm in the pure curved segment between the two loading points, which is shown in Figure 20.

#### 4.3.3. Results and Discussion

**Figure 21:**
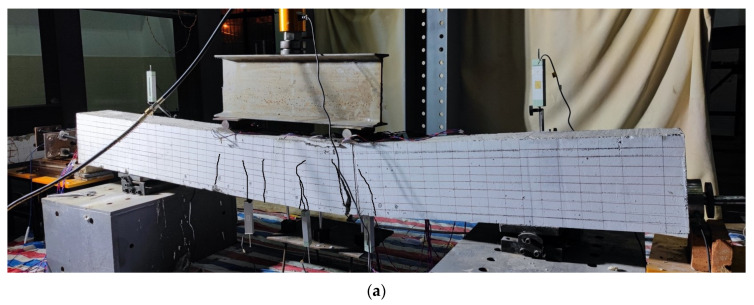

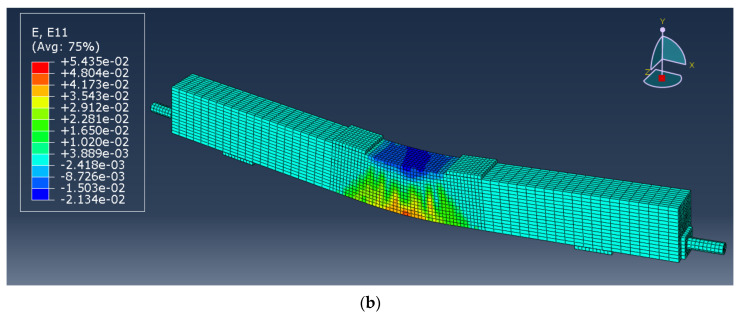
The PC beam after loading. (**a**) Experimental diagram; (**b**) FEM diagram.

Figure 21a,b, respectively, show the experimental diagram and FEM diagram of the PC beam after loading.

As can be seen from the figure, when the PC beam reaches its ultimate bearing capacity, the concrete on the top of the beam is crushed, and the cracks in the tension area develop to the position of 4/5 beam height.

**Figure 22:**
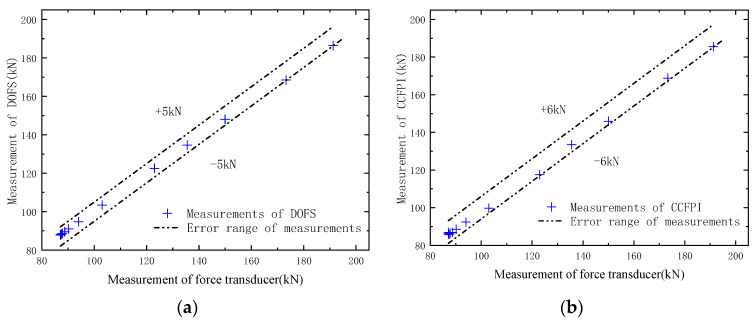
Comparison of the cable force measured by the OECS-CFRP cable and force transducer under different loads. (**a**) Measurements of DOFS; (**b**) measurements of CCFPI.

Figure 22 and Table 7 show the comparison between the cable force measured by the OECS-CFRP cable and that measured by the force transducer. It can be seen from Figure 22a that the error between the measurements of DOFS and that of the force transducer is within ±5 kN, and the relative error is 2.62%. The error between the measurements of CCFPI and that of the force transducer is within ±6 kN, as shown in Figure 22b, and the relative error is 3.14%. The results show that the OECS-CFRP cable can accurately perceive the cable force during the whole loading process of the prestressed concrete beam.

**Figure 23:**
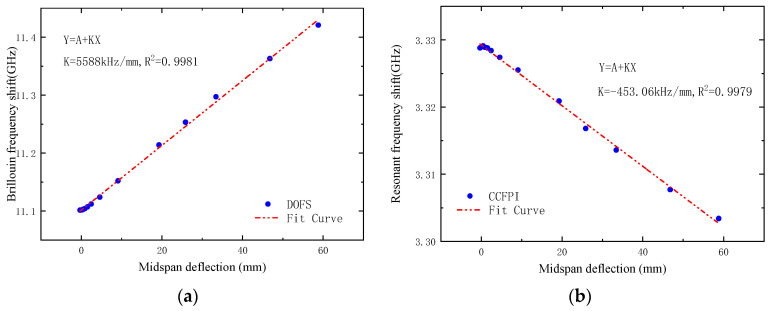
Relationship between frequency shift of DOFS/CCFPI and midspan deflection. (**a**) DOFS; (**b**) CCFPI.

Figure 23a,b, respectively, show the relationship between the frequency shift of DOFS and CCFPI and midspan deflection. It can be seen that the frequency shift of DOFS and CCFPI has a good linear relationship with the midspan deflection of the test beam. The deflection sensitivities of DOFS and CCFPI are, respectively, 5.588 MHz/mm and −453.1 kHz/mm, and the square of linear correlation coefficients are both above 0.997.

**Figure 24:**
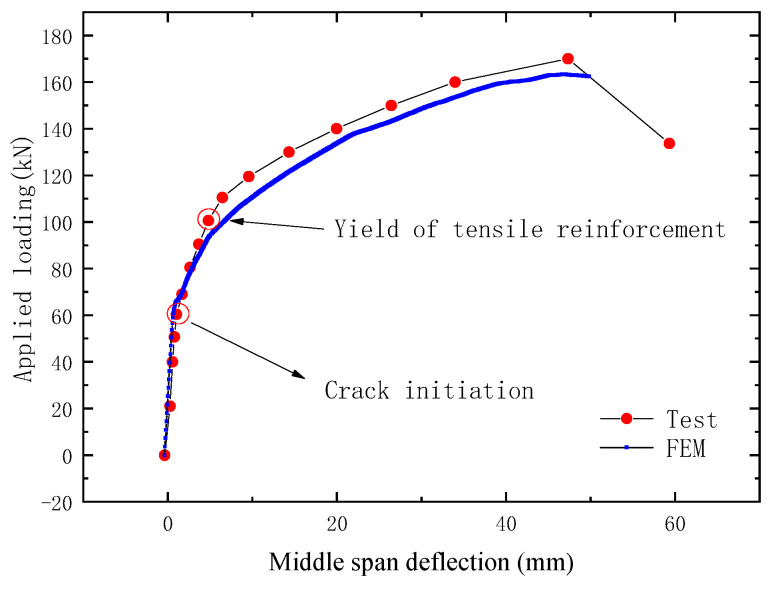
Variation of midspan deflection of test beam under different loads.

The load versus deflection curve of the test beam is shown in Figure 24. The horizontal coordinate is the midspan deflection of the PC beam, and the vertical coordinate is the vertical load of four-point bending tests. It can be seen from the figure that the test curve is in good agreement with the FEM curve. With the load increasing gradually, the midspan deflection of the beam also increases. The tensile stress was borne by the CFRP cable, the bottom tensile reinforcements, and the concrete in the tension area of the test beam in the initial stage; thus, the deflection of the beam increased slowly. When the load increased to 70 kN, cracks were generated in the tensile area of the pure bend section of the test beam, and the tensile stress was borne by the CFRP cable and the bottom tensile reinforcements. Thus the deflection increased faster. However, when the load increased to 100 kN, the bottom tensile reinforcements yielded, and the tensile stress increment was borne by the CFRP cable only. Thus the deflection increased faster again. The test results show that the OECS-CFRP cable can monitor the midspan deflection of the prestressed beam so as to obtain the stiffness degradation of the prestressed beam under different loads.

**Figure 25:**
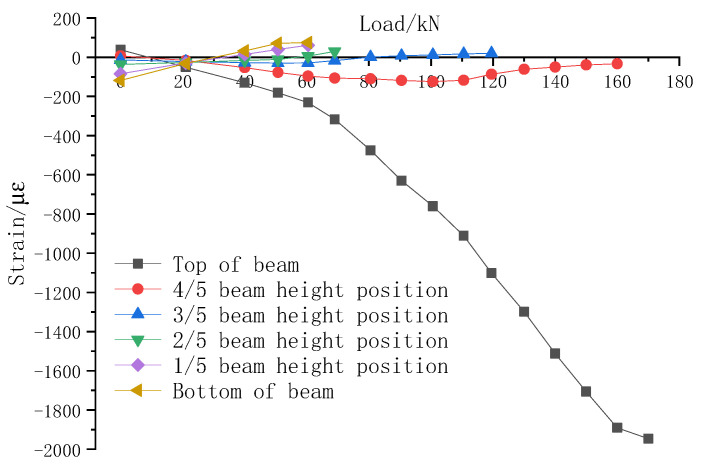
Variation of midspan concrete strain of test beam under different loads.

Figure 25 shows the variation of midspan concrete strain of the test beam under different loads. It can be seen that the strain of concrete at the bottom of the PC beam and top of the PC beam were 75 με and −231 με, respectively, when the cracks appeared along the PC beam.

## 5. Conclusions

This paper proposed a novel smart CFRP cable based on optical–electrical co-sensing for full-process prestress monitoring of structures. Then, the sensing performance of the OECS-CFRP cable was characterized by serious experiments. Finally, the OECS-CFRP cable was used for the prestress monitoring of an unbonded prestressed RC beam. The results show that the DOFS and CCFPI in the OECS-CFRP cable both have good linearity of 2.64% FS and 7.14% FS, repeatability of 1.25% FS and 4.28% FS, and hysteresis of 1.43% FS and 4.34% FS. Compared with DOFS, CCFPI has relatively lower accuracy and resolution but enough measurement range to tolerate large strain when the CFRP bars of the cable is stretched to burst. In the loading test of the unbonded prestressed RC beam, the OECS-CFRP cable can effectively monitor the cable force and the midspan defection of the beam. Thus, the OECS-CFRP cable can obtain the stiffness degradation of the prestressed beam under different loads.

As a next step, a comparison with already published works will be discussed. Further, the behavior of the OECS-CFRP cable under mechanical and environmental cyclic loading should be studied. Then, several OECS-CFRP cables will be informed by the anchor tray to form a large tonnage cable. The corresponding sensing and mechanical properties of the large tonnage cable also need to be studied. Finally, the proposed OECS-CFRP cable is expected to solve the corrosion problem and the whole process of monitoring prestressed tendons in PC structures under harsh environments.

## Figures and Tables

**Table 1:** The main static performance indexes of the OECS-CFRP cable.

Sensor	Linearity	Hysteresis	Repeatability	Resolution/kN
DOFS-C1	2.64%	1.24%	0.68%	0.073
DOFS-C2	2.60%	1.43%	1.25%	0.072
CCFPI-C1	5.99%	4.34%	2.65%	0.986
CCFPI-C2	7.14%	2.47%	4.28%	1.217

**Table 2:** Mechanical properties of OECS-CFRP cable.

E_ca_ (GPa)	P_ca_ (kN)	σ_ca_ (MPa)
163.83	178	1676

**Table 3:** Comparing methods for monitoring prestressing.

Methods	Sensitivity to Changes in the Prestressing Force	Force Distribution along the Structure	Accuracy	Effects of Environmental Temperature	Cost		Applicability to CFRP Cable
					Sensor	Instrument	
PZT-interface	High (locally)	Local but only at anchorage	Low	High	High	High	Not explored
Acoustoelastic methods	Low	Not feasible, global	Medium	Not explored	None	High	Not explored
Elasto-magnetic methods	High	Feasible, local	High	High (can be calibrated)	High	High	Unfeasible
FBG sensors	High	Feasible, local	High	High (can be compensated)	High	Medium	Cable normal use phase only
DOFS	High	Feasible, Distributed	High	High (can be compensated)	Low	High	Cable normal use phase only
CC-FPI	Medium	Feasible, local	Medium	High (can be compensated)	Low	Medium	Cable normal use phase and failure stage
OECS-CFRP cable	High	Feasible	High	High (can be compensated)	Low	High	Cable normal use phase and failure stage

**Table 4:** Mechanical properties of concrete.

Age (d)	f_cu,k_ (MPa)	f_ck_ (MPa)	E_c_ (MPa)	υ
28	42.2	26.9	3.26 × 10^4^	0.31

**Table 5:** Mechanical properties of steel reinforcement.

Diameter (mm)	f_yk_ (MPa)	f_stk_ (MPa)	E_s_ (MPa)
C10	476	644	2.10 × 10^5^

**Table 6:** The performance parameters of the sensors.

Sensors	Frequency	Range	Sensitivity	Resolution	Accuracy
Dial indicator (YHD-100)	1 Hz	±50 mm	0.2 mv/mm	0.001 mm	0.01 mm
Force transducer (BLR-1)	1 Hz	50 t	0.01069 mv/kN	0.001 kN	±0.5%

**Table 7:** The comparison errors between the prestress force measurements obtained from the smart CFRP cable and force transducer.

Vertical Load (kN)	Force Transducer (kN)	DOFS			CCFPI		
		Values (kN)	Absolute Error (kN)	Relative Error (%)	Values (kN)	Absolute Error (kN)	Relative Error (%)
0	87.1	87.8	0.7	0.80	86.9	0.2	0.21
21	87.1	87.9	0.8	0.92	86.1	1.0	1.15
40	87.4	88.2	0.8	0.92	85.8	1.6	1.83
60.4	87.8	88.6	0.8	0.91	86.5	1.3	1.48
69	88.7	89.5	0.8	0.90	86.9	0.8	0.90
80.5	90.2	91.0	0.8	0.89	88.5	1.7	1.88
100.6	94	94.7	0.7	0.74	92.4	1.6	1.70
119.5	103	103.4	0.4	0.39	99.7	3.3	3.20
140	123	122.6	0.4	0.33	117.6	5.4	4.39
150	135.5	134.6	0.7	0.52	133.5	2.0	1.48
160	150	148.1	1.7	1.13	145.9	4.1	2.73
170	173.3	168.5	4.6	2.65	168.8	4.5	2.60
133.7	191.3	186.4	4.7	2.46	185.5	5.8	3.03

## Data Availability

Not applicable.
